
JOURNAL INFORMATION
==============================
NLM Title Abbreviation: Wirtschaftsdienst
Journal ID: Wirtschaftsdienst
NLM Journal ID: 101086612

Wirtschaftsdienst (Hamburg, Germany : 1949)

ISSN: 0043-6275

ARTICLE INFORMATION
==============================
PMCID: PMC8218270
PMID: 34176990
DOI: 10.1007/s10273-021-2947-9
Article ID: 2947
Article version: 1
Subjects: Ökonomische Trends

Konjunkturschlaglicht: Zeichen der Erholung im deutschen Außenhandel

Economic Headline: Signs of Recovery in German Foreign Trade

Wolf André wolf@hwwi.org

grid.435054.3 0000 0001 2227 8589 Hamburgisches WeltWirtschaftsInstitut (HWWI), Oberhafenstraße 1, 20097 Hamburg, Deutschland
Electronic publication date: 2021 Jun 22
Print publication date: 2021
Volume: 101
Issue: 6
First page: 487
Last page: 488
Copyright: © Der/die Autor:in(nen) 2021
License: Open Access: Dieser Artikel wird unter der Creative Commons Namensnennung 4.0 International Lizenz veröffentlicht (https://creativecommons.org/licenses/by/4.0/deed.de).
Open Access wird durch die ZBW — Leibniz-Informationszentrum Wirtschaft gefördert.
License URL: https://creativecommons.org/licenses/by/4.0/

issue-copyright-statement: © The Author(s) 2021

==============================

Die wirtschaftlichen Implikationen von COVID-19 haben den Welthandel seit Ausbruch der Pandemie maßgeblich geprägt. Im 2. Quartal 2020 brach der grenzüberschreitende Warenaustausch wertmäßig um 21,4 % gegenüber dem Vorjahresquartal ein (WTO, 2021), der stärkste Quartalsrückgang seit der Finanzkrise 2009. Das Ausmaß des Absturzes ist über das Zusammenwirken mehrerer Schockwirkungen erklärbar. Angebotsseitig hat die verordnete Schließung von Produktionsanlagen zu Störungen in den internationalen Lieferketten der Warenproduktion geführt. Während diese Schließungen nur temporär und räumlich vereinzelt auftraten, war der nachfrageseitige Schock globaler Natur. Beschäftigungsverluste und um sich greifende Unsicherheit führten zur Konsumzurückhaltung im Hinblick auf größere Anschaffungen (Baldwin und Tomiura, 2020). So sank vor allem die Nachfrage nach teuren langlebigen Gebrauchsgütern, deren Produktionswege durch ein hohes Maß an internationaler Arbeitsteilung gekennzeichnet sind. Umso erstaunlicher erscheint vor diesem Hintergrund die relativ schnelle Erholung des internationalen Handels im weiteren Jahresverlauf. Bereits im 3. Quartal 2020 wurde der Handelswert des 1. Quartals übertroffen, im 4. Quartal schließlich sogar ein positives Wachstum gegenüber dem Vorjahresquartal realisiert. Die deutsche Wirtschaft ist angesichts ihres hohen Offenheitsgrades von diesen Turbulenzen besonders stark betroffen. Im Zuge der Pandemie sank der deutsche Außenhandelssaldo 2020 gegenüber 2019 von 224 Mrd. auf 180 Mrd. Euro. Anders als auf globaler Ebene verlief bei den deutschen Exporten der Erholungsprozess zu Jahresende eher schleppend (vgl. Abbildung 1). Die zuletzt für März 2021 veröffentlichten Monatszahlen belegen jedoch wieder einen klaren Aufwärtstrend bei den deutschen Ausfuhren, und das nicht nur gegenüber der besonderen Situation im Vorjahr: Der Exportwert (126,7 Mrd. Euro) überstieg auch den gemessenen Wert für März 2019 (118,2 Mrd. Euro) (Destatis, 2021).

Ein Blick auf die Strukturfaktoren hinter dieser Statistik liefert ein differenziertes Bild. Die für die deutschen Ein- und Ausfuhren zu beobachtende Negativdynamik setzte zu einem relativ frühen Zeitpunkt im Jahr 2020 ein, bereits vor den ersten pandemiebedingten Einschränkungen im Inland. Die Tatsache, dass zunächst China als Deutschlands wichtigster Handelspartner im Import- und zweitwichtigster Partner im Exportbereich ein Ausbreitungsschwerpunkt von COVID-19 war, schlug sich entsprechend deutlich in den Handelszahlen nieder. Im Januar und Februar 2020 ergab sich jeweils ein signifikanter Rückgang der deutschen Exporte in die Volksrepublik im Vergleich zu den Vormonaten um -12,2 % bzw. -7,6 %[1] (Destatis, 2021). Die Importe aus China erlebten dagegen nur im Februar einen starken Einbruch, dem aber bereits im April 2020 wieder eine deutliche Erholung gegenüberstand. In der Quartalsbetrachtung zeigt sich, dass der Rückgang der deutschen Ausfuhren in der Frühphase geografisch allerdings schon durchaus breitgefächert war. So schrumpften im 1. Quartal 2020 im Handel bei allen fünf wichtigsten Abnehmerländern die deutschen Exporte gegenüber dem Vorjahresquartal (vgl. Tabelle 1). Dies ist allerdings nicht ausschließlich im Zusammenhang mit frühzeitigen Pandemiefolgen zu sehen. So kommen im Fall der besonders stark zurückgehenden Exporte nach Großbritannien auch Folgen des Brexit zum Tragen, der Einbruch setzte hier bereits im 2. Quartal 2019 ein. Das 2. Quartal 2020 war dann infolge der globalen Virusausbreitung durch eine Schockwirkung größeren Ausmaßes geprägt, die sich auf breiter Front in den deutschen Exporten niederschlug. Auch diese Entwicklung verlief geografisch nicht homogen. Während der Absatz deutscher Waren im europäischen Ausland und in den USA gegenüber dem Vorjahresquartal stark einbrach, wurde für die Exporte Richtung China nur noch ein geringer Rückgang gemessen. Zugleich stiegen die Einfuhren

1 Hier und im Folgenden: Veränderungsraten auf Euro-Basis (nominal).

Abbildung 1

**Ein- und Ausfuhren Deutschlands: Differenz zum Vorjahresmonat**

Quelle: Destatis (2021).

Tabelle 1
**Wert der deutschen Ausfuhren nach Handelspartnern**

| | Status quo (Mrd. Euro) | Entwicklung: Vergleich zu Vorjahresquartalen (%) | | | | |
|---|---|---|---|---|---|---|
| Handels-partner | 1. Quartal 2021 | 1. Quartal 2019-2020 | 2. Quartal 2019-2020 | 3. Quartal 2019-2020 | 4. Quartal 2019-2020 | 1. Quartal 2020-2021 |
| USA | 29,08 | -1,34 | -31,25 | -14,81 | -2,60 | 1,07 |
| China | 26,32 | -8,28 | -4,36 | 3,00 | 8,51 | 22,31 |
| Frankreich | 25,53 | -8,39 | -30,74 | -12,15 | -7,75 | -0,09 |
| Niederlande | 24,3 | -6,13 | -16,16 | -8,63 | 0,15 | 8,81 |
| Großbritannien | 16,25 | -13,96 | -35,62 | -10,30 | -3,84 | -17,43 |

Quelle: Destatis (2021).

aus China bereits wieder spürbar. Die schnellen Eindämmungserfolge im Ursprungsland der Pandemie haben sich also offenkundig stabilisierend auf den deutschen Außenhandel ausgewirkt. Im 2. Halbjahr 2020 erwies sich der Chinahandel dann auch als entscheidender Motor der Erholung. Erst recht gilt dies für das 1. Quartal 2021. Die im Zuge der aufgetretenen Virusvarianten wieder ansteigende Ansteckungsinzidenz im europäischen Raum hat eine deutliche Erholung der Intra-EU-Exporte gegenüber dem Vorjahresquartal verhindert. Auch die Ausfuhren in die USA zeigten zunächst noch keinen signifikanten Aufwärtstrend. Einen klaren länderübergreifenden Schub erhielt der deutsche Außenhandel dann erst im März 2021.

Eine Untersuchung der jüngsten Handelsentwicklung nach Gütergruppen lässt ebenfalls auffällige, im Zusammenhang mit der geografischen Verteilung von Pandemiemaßnahmen stehende Muster erkennen. So verzeichneten unter den wichtigsten deutschen Exportgütern[2] im 1. Quartal 2020 zunächst nur Kraftwagen und Kraftwagenteile (-10,6 %) sowie in schwächerem Ausmaß Metallerzeugnisse (-5,5 %) und Maschinen (-5,2 %) einen spürbaren Rückgang im Exportwert gegenüber dem Vorjahresquartal, d. h. Gütergruppen, bei denen Formen von vertikaler Verflechtung entlang der Lieferkette zwischen deutschen und chinesischen Standorten besonders stark ausgeprägt sind. Auch im 2. Quartal 2020 wurden die stärksten Einbrüche für diese Gütergruppen beobachtet, der Exportwert im Kfz-Bereich sank um nahezu 50 % gegenüber dem 2. Quartal 2019. Zugleich schrumpften die Ausfuhren nun aber auch in allen übrigen Gruppen, mit Ausnahme der pharmazeutischen Erzeugnisse (+6,2 %), ein Spiegel der global gewordenen Pandemiesituation. Im 4. Quartal 2020 zeigten sich dann wiederum güterspezifische Erholungsmuster. Positive Impulse für die deutschen Exporte gingen hier zunächst vorrangig von den Segmenten Metalle (+5,7 % gegenüber Vorjahresquartal), Elektrische Ausrüstungen (+4,8 %) und Kfz (+4,5 %) aus. Auch für die im jüngsten Berichtsquartal beobachtbare Erholung sind diese Produkte maßgeblich. Unter den zehn wichtigsten Gütergruppen ist in Quartalsbetrachtung nun mehrheitlich wieder ein Positivwachstum zu konstatieren, mit dem Bereich Sonstiger Fahrzeugbau als Negativausreißer (-26,3 %). Im jüngsten Berichtsmonat März gilt dies mit Ausnahme des letztgenannten Segments mittlerweile für sämtliche industrielle Waren.

2 Destatis, 2021, Aggregationsebene: GP2019 2-Steller.

Die Erwartungen in Bezug auf den Außenhandel im 2. Quartal 2021 sind in Teilen noch durch die angespannte Pandemielage in Europa während des Monats April belastet. Die Nachfrage Chinas wird vor diesem Hintergrund ein wichtiger Motor bleiben: Die OECD erwartet in ihren aktuellen Schätzungen für 2021 ein kräftiges Wachstum von +8,5 % für das chinesische Bruttoinlandsprodukt (BIP) (OECD, 2021). Hinzu kommt aber nun auch die wirtschaftliche Entwicklung in den USA als zweiter Wachstumsimpuls: Das von der neuen US-Administration verabschiedete Konjunkturpaket hat im Zusammenhang mit sinkenden Corona-Fallzahlen bereits im 1. Quartal 2021 zu einer überraschend starken Erholung der US-Wirtschaft beigetragen. Für das US-BIP prognostiziert die OECD bezogen auf das Gesamtjahr einen ebenfalls beeindruckenden Anstieg von 6,9 %. Im weiteren Jahresverlauf sollte dann auch der Intra-EU-Handel wieder verstärkt Fahrt aufnehmen. Entscheidende Voraussetzung ist, dass das mittlerweile deutlich gestiegene Impftempo in den EU-Ländern aufrechterhalten wird und es zu keiner Ausbreitung neuer impfresistenter Virusvarianten kommt. Ein weiterer im Auge zu behaltender Risikofaktor für die deutsche Exportentwicklung 2021 stellt eine Fortsetzung der gegenwärtigen nachfragebedingten Preisanstiege an den internationalen Rohstoffmärkten dar. Diese könnte die deutsche Wirtschaft als Rohstoffimporteur kostenseitig im besonderen Maße belasten (Wellenreuther, 2021).

André Wolf
wolf@hwwi.org


Literatur

Baldwin, R., und E. Tomiura, E. (2020), Thinking ahead about the trade impact of COVID-19. Economics in the Time of COVID-19, 59.
Destatis (2021), Außenhandel. Statistik der Ein- und Ausfuhren, Statistisches Bundesamt, https://www.destatis.de/DE/Themen/Wirtschaft/Aussenhandel/Jnhalt.html (4. Juni 2021).
OECD (2021), OECD Economic outlook, Mai, https://www.oecd.org/economic-outlook/may-2021/ (4. Juni 2021).
Wellenreuther C Konjunkturschlaglicht: Rohstoffpreise unter dem Einfluss von COVID-19 Wirtschaftsdienst 2021 101 2 147 148 10.1007/s10273-021-2858-9 33642651 PMC7896181
WTO (2021), Total merchandise exports quarterly, https://data.wto.org/ (4. Juni 2021).
